# A machine learning model based on CHAT-23 for early screening of autism in Chinese children

**DOI:** 10.3389/fped.2024.1400110

**Published:** 2024-09-10

**Authors:** Hengyang Lu, Heng Zhang, Yi Zhong, Xiang-Yu Meng, Meng-Fei Zhang, Ting Qiu

**Affiliations:** ^1^ School of Artificial Intelligence and Computer Science, Jiangnan University, Wuxi, China; ^2^ Engineering Research Center of Intelligent Technology for Healthcare, Ministry of Education, Wuxi, China; ^3^ Department of Child Health Care, Affiliated Women’s Hospital of Jiangnan University, Wuxi, China

**Keywords:** autism spectrum disorder, CHAT-23, early screening, feature engineering, machine learning, Chinese children

## Abstract

**Introduction:**

Autism spectrum disorder (ASD) is a neurodevelopmental condition that significantly impacts the mental, emotional, and social development of children. Early screening for ASD typically involves the use of a series of questionnaires. With answers to these questionnaires, healthcare professionals can identify whether a child is at risk for developing ASD and refer them for further evaluation and diagnosis. CHAT-23 is an effective and widely used screening test in China for the early screening of ASD, which contains 23 different kinds of questions.

**Methods:**

We have collected clinical data from Wuxi, China. All the questions of CHAT-23 are regarded as different kinds of features for building machine learning models. We introduce machine learning methods into ASD screening, using the Max-Relevance and Min-Redundancy (mRMR) feature selection method to analyze the most important questions among all 23 from the collected CHAT-23 questionnaires. Seven mainstream supervised machine learning models were built and experiments were conducted.

**Results:**

Among the seven supervised machine learning models evaluated, the best-performing model achieved a sensitivity of 0.909 and a specificity of 0.922 when the number of features was reduced to 9. This demonstrates the model's ability to accurately identify children for ASD with high precision, even with a more concise set of features.

**Discussion:**

Our study focuses on the health of Chinese children, introducing machine learning methods to provide more accurate and effective early screening tests for autism. This approach not only enhances the early detection of ASD but also helps in refining the CHAT-23 questionnaire by identifying the most relevant questions for the diagnosis process.

## 1 Introduction

Autism spectrum disorder (ASD) is a neurodevelopmental disorder usually manifested by behavioral and social deficits (1). The prevalence of autism has been increasing over the years, with varying estimates depending on the region and population studied. Statistics indicate that over 1.5% of children in the United States, or approximately one in every 54 children, are diagnosed with ASD. However, the prevalence of autism in China is estimated to be lower than 1%. This difference may be due to the underrepresentation of the mainstream school population in the statistical data and the need for more up-to-date research to assess the prevalence of autism in China accurately (2, 3). Matson et al. conducted research on cross-cultural autism behavior and also showed that children with autism have different behavioral characteristics and severity due to regional differences and cultural backgrounds (4).

Infancy is a critical period of plasticity for brain development (5, 6). The primary objective of early intervention is to make full use of brain plasticity during infancy, enhance brain development induce behavioral changes, and ultimately reduce the impact of the disorder in children with ASD. Based on the characteristics of ASD in 8-year-olds, it has been found that the diagnostic accuracy of children under 36 months of age exceeds that of children over 36 months of age. Some studies use different age groups as controls, finding that younger children, compared to adolescents, provide more stable and accurate results for early diagnosis (7, 8). Those findings highlight the importance of early screening for autism. Early screening allows for timely intervention to help children receive treatment for autism (9). There are two main lines for early screening of autism, including Scale-based methods, and machine learning-based methods.

Scale-based early screening tests for ASD can be classified into two levels: the level 1 ASD screening test and the level 2 ASD screening test. Examples include the Modified Checklist for Autism in Toddlers (M-CHAT) (10), the Quantitative Checklist for Autism in Toddlers (Q-CHAT) (11), and the Checklist for Autism in Toddlers (CHAT-23) (12). On the other hand, Level 2 ASD screening tests are intended for children at high risk for ASD, such as the Infant-Toddler Checklist (ITC) (13), the Baby and Infant Screen for Children with aUtIsm Traits (BISCUIT) (14), and the Systematic Observation of Red Flags (SORF) (15). Among various screening tests, the CHAT-23 serves as a Level 1 screening test for ASD used in Chinese children. CHAT-23 is a scale screening test consisting of a parental questionnaire and a physician’s observation of the children’s behaviors. This test has significant value in early screening as it assesses a child’s social and behavioral performance, enabling the recognition of potential signs of autism (16). For example, an online screening system utilizing telemedicine technology was developed for ASD by using CHAT-23 (17).

Machine learning based ASD methods require training on data, which can be in the form of questionnaires or image data. Machine learning is a technique that automatically analyzes data to obtain patterns and uses them to make predictions about unknown data (18). Benefiting from the ability to learn from existing data, machine learning has become a popular way for ASD (19). Depending on the type of data used for machine learning, there are two main types of machine learning based early screening methods, one is the Scale-based method, and the other is image-based method. Scale-based methods have solid practical experience from experts and can provide strong evidence for the machine learning model. For example, based on the scale information collected using the Q-CHAT, a range of machine learning models including Logistic Regression, Support Vector Machine, Naive Bayes, Random Forest, and K-Nearest Neighbors have been used to predict autism in toddlers (20). Using logistic regression and decision tree algorithm, Duda et al. found that 7 key items were selected from all items in the Autism Diagnostic Interview-Revised (ADI-R) to build an accurate model for classifying children with autism (21). Multiple machine learning methods have been effectively demonstrated to differentiate between Autism Spectrum Disorder (ASD) and Attention-Deficit/Hyperactivity Disorder (ADHD) (22). Some unsupervised clustering machine learning methods also have been employed for modeling data related to Autism Spectrum Disorder (ASD), and their results have proven to help facilitate autism diagnosis (23). Siddiqui et al. achieved a high classification accuracy for children with ASD by utilizing monitoring devices to acquire behavioral gesture information. They employed models such as K-Nearest Neighbors and Random Forest to perform the classification (24). Biomarkers based on brain imaging as input features for machine learning techniques can provide objective evidence for autism classification. Functional magnetic resonance imaging (fMRI) data combined with machine learning models has also been utilized to assist in diagnosing ASD (25). There also exists mixed data used for modeling machine learning. Abbas et al. create low-cost and accurate autism screening tests using parent report questionnaires and home videos of children (26).

Several studies show that ASD is closely connected with other diseases. Precenzano et al. demonstrated that electroencephalographic abnormalities are typically associated with more severe forms of ASD (27). Rossi et al. reported that nearly half of children with ASD, indicated by Subclinical Electroencephalographic Abnormalities, even without epilepsy, show abnormal development within the first year, with epilepsy and intellectual disability (28). Early diagnosis of ASD can be beneficial, as it allows for treatment before the condition worsens, potentially reducing costs and promoting family life. Operto et al.’s report showed that ASD can be treated at an earlier stage by means of cheaper intervention, such as speech therapy, psychomotor therapy, occupational therapy, etc. As grow older, treatment may have to consider drug therapy, and the amount of drugs used increases with age (29). Therefore, considering the importance of early screening, we believe that highly efficient machine learning-based methods for early autism screening should be considered. Several machine learning based methods for early screening validate their effectiveness on American children (26, 30, 31). However, there is still a vacant study on Chinese children. Thus, we first collected questionnaire data from age-appropriate children and processed the raw data through a series of preprocessing steps to make it suitable for modeling. We then built seven different machine learning models using above data. From all the models, we identified the most appropriate one to assist in diagnosing ASD. In conclusion, our work and main contributions include:

(1) We built machine learning models by applying data from Chinese children.
(2) We applied mRMR feature engineering to automatically acquire a set of features for better construction of machine learning models.
(3) We modeled seven different machine learning models, and compared the prediction performance of these models based on collected question data, identifying the best-performing machine learning model.
(4) We performed feature engineering upon the original 23-dimensional question features, and rank these questions according to their importance. Finally, the LGBM model achieved the highest specificity and sensitivity of 0.922 and 0.909.

## 2 Materials and methods

### 2.1 Materials and participants

**Figure 1:**
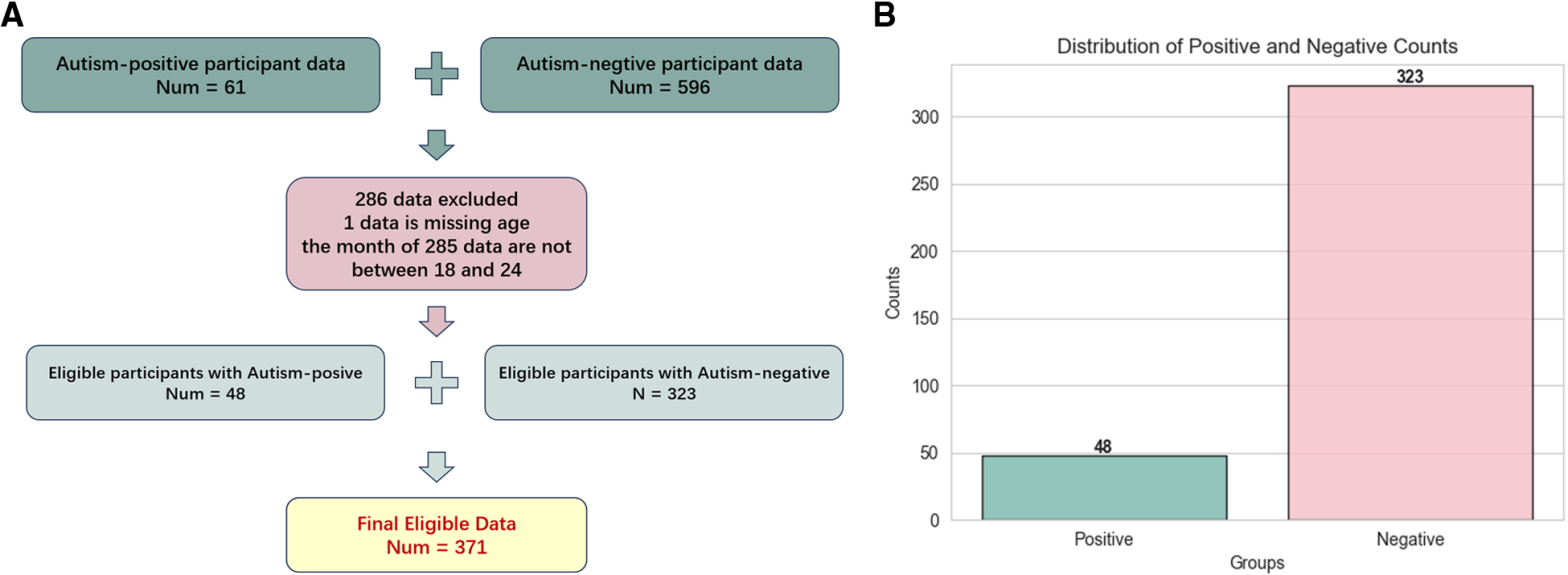
Figure 1A is the flow chart of participants selection, and Figure 1B is the distribution of positive and negative results for autism in 371 eligible questionnaires.

This study uses data collected from the Affiliated Hospital of Jiangnan University, ranging from January 4th, 2022 to September 28th, 2022. Participants selection flow is as Figure 1A shows. According to the practice of CHAT-23, the applicable age is 18 to 24 months, we exclude those data whose month age does not meet this range. We also exclude those questionnaires with missing information. This participant selection flow obtained 371 copies of the children’s CHAT-23 scale information. According to statistics, the average age of children is (20.27 ± 2.68) months. As shown in Figure 1B, in all cases, 48 people were diagnosed as autism positive and 323 were negative, which is an important basis for our research.

### 2.2 Study design

**Figure 2:**
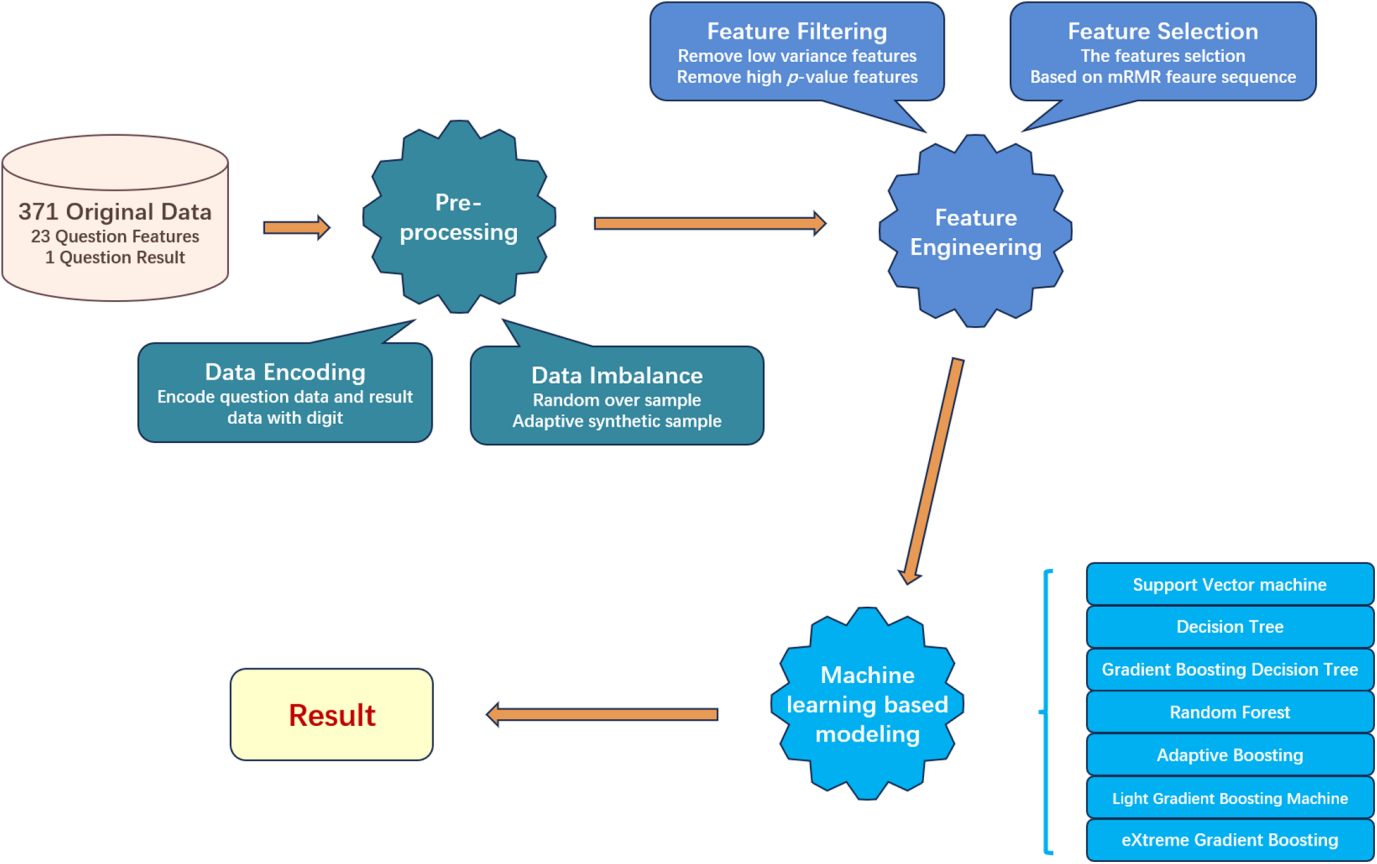
General workflow of the machine learning based mRMR with feature engineering.

After we got eligible data, three main processes were divided into five minor steps designed to process and screen important data. The overall workflow is as Figure 2 shows. The three main processes are preprocessing, feature engineering, and machine learning model building, each focusing respectively on data, features, and models. The first step is data encoding. In this step, we encode the text information collected in the questionnaire into a digital format, so machine learning models can process that. Next step, we split the dataset into training and testing sets. As the negative and positive samples are imbalanced for the training set, we will use resampling techniques to balance the numbers of samples from each class, to mitigate the impact of data imbalance on model construction. In the third step, we conduct feature filtering based on statistical methods to reduce the original feature dimension. The next step is feature selection, in which we used mRMR to further find out the most relevant features to help the model build. In the last step, we build seven machine learning models to compare and evaluate the experimental results.

### 2.3 Data encoding

To encode the text data from the CHAT-23 questionnaires, we follow the experience introduced in Vakadakar’s work (32). Since the collected questionnaire data is in text form, for each CHAT-23 question section, we set four different frequency words to record the test subject’s information, which is never, occasionally, sometimes, and often. We encode them as 0, 1, 2, 3 respectively. For the diagnosis results, we use 0 to represent negative, and we use 1 to represent positive respectively. In the end, we get the normalized 23-question characteristics.

### 2.4 Data imbalance

We found there exists a clear imbalance in the experimental data, with 323 negatives vs. 48 positives. Since there are significantly more negative samples than positive samples, if we do not process this phenomenon, the machine learning based model may prefer to remember the characteristics of the negative samples but despise the positive sample features. The data imbalance would result in the model having a high prediction success rate for negative samples, while the positive sample prediction effect is poor. This will lead to a catastrophic decrease in sensitivity.

To balance the impact of negative and positive input data in the model, we use a combination of up-and-down sampling techniques. We first use down-sampling technology, which randomly deletes negative samples to reduce the number of negative samples. We set the parameters of the sampling strategy to 0.3, the negative sample data is randomly retained at 160. However, if we use too aggressive down-sampling strategy, we are likely to lose the information of negative samples, so we use a combination of up-sampling technology. We copy some positive samples, and set the parameters of the up-sampling strategy to 0.5, so we can get 80 positive samples. The resampled data is fed into the model, which balances the model’s ability to learn from both positive and negative samples.

### 2.5 Feature filtering

Not all question features from CHAT-23 are useful for machine learning models to predict outcomes correctly, so we use statistical-based filtering to find and remove these less important features. We use variance and p-value as two criteria for filtering.

First, we calculate the variance value of each question feature of all instances. The larger the variance, the greater the change in the value range of the feature in the sample, which means that the feature has a stronger ability to distinguish whether the target is finally positive. In this way, the characteristic is more conducive to building a model. Among them, those features with small variance are similar to most people, regardless of negative or positive testers, and their values on these features are similar, so this feature is relatively secondary to the discriminant result. There are two features with variance below 0.1, which correspond to Q12 and Q16. Through the above steps, we removed the features with variance below 0.1 from the original 23 question features, while retaining the remaining 21 features.

Next, we utilize the chi-square method to calculate the p-value for each feature, taking into consideration the correlation between the feature and the diagnosis (4). We regard features with a *p*-value less than 0.05 as features that are important to the diagnosis result. Thus, we remove all features with a *p*-value greater than 0.05 and finally retain 15 features, and the results are listed in Table 1. The corresponding questions to removed features include Q1, Q3, Q4, Q10, Q11, Q18.

**Table 1:** 15 Features are selected based on feature filtering.

Rank	Feature	*p*-value*
1	Q20	$5.90\times 10^{-12}$
2	Q5	$1.30\times 10^{-10}$
3	Q21	$1.62\times 10^{-5}$
4	Q7	$2.12\times 10^{-5}$
5	Q13	$5.22\times 10^{-4}$
6	Q17	$9.64\times 10^{-4}$
7	Q6	$1.96\times 10^{-4}$
8	Q23	$2.65\times 10^{-4}$
9	Q9	$5.49\times 10^{-4}$
10	Q15	$1.37\times 10^{-3}$
11	Q14	$1.61\times 10^{-3}$
12	Q22	$1.73\times 10^{-3}$
13	Q19	$3.12\times 10^{-3}$
14	Q2	$1.64\times 10^{-2}$
15	Q8	$1.74\times 10^{-2}$

* This table displays features with the highest correlation with labels, whose *p*-values are less than 0.05.

### 2.6 Feature selection

A suitable feature selection not only improves the accuracy of machine learning methods’ predictions but also reflects which features are more important for predicting negative and positive outcomes. mRMR can discover those features that are most relevant to the diagnostic results among all features and have the least redundancy between each feature. Thus, we use mRMR to select features sequentially. In mRMR, the MIQ parameter calculates the correlation between two features through mutual information and measures the degree of confusion between multiple feature groups through information entropy. After setting different weights for the calculated correlation and difference, we can get the sequence of features, and the higher the feature is considered as the more important feature. The sequence of this feature will be incrementally used as input to the model to train different machine-learning models. The feature sequences discovered by the mRMR method are displayed in Table 2.

**Table 2:** The sequence of 15 question features is based on mRMR.

Rank	Question number	Question descriptions in CHAT-23
1	Q21	Does your child understand what people say?
2	Q6	Does your child ever use his/her index finger to point, to ask for something?
3	Q9	Does your child ever bring objects over to you (parent) to show you something?
4	Q13	Does your child imitate you? (e.g., you make a face; will your child imitate it?)
5	Q7	Does your child ever use his/her index finger to point, to indicate interest in something?
6	Q14	Does your child respond to his/her name when you call?
7	Q5	Does your child ever pretend, for example, to talk on the phone take care of dolls, or pretend other things?
8	Q15	If you point at a toy across the room, does your child look at it?
9	Q17	Does your child look at things you are looking at?
10	Q20	Have you ever wondered if your child is deaf?
11	Q2	Does your child take an interest in other children?
12	Q23	Does your child look at your face to check your reaction when faced with something unfamiliar?
13	Q8	Can your child play properly with small toys (e.g., cars or bricks) without just mouthing, fiddling, or dropping them?
14	Q19	Does your child try to attract your attention to his/her own activity?
15	Q22	Does your child sometimes stare at nothing or wander with no purpose?

### 2.7 Machine learning based models

We choose 7 widely used supervised machine learning models to perform primary screening for autism, including two non-ensemble learning models: Support Vector Machine (SVM), and Decision Tree (DT), as well as four ensemble learning models: Gradient Boosting Decision Tree (GBDT), Light Gradient Boosting Machine (LGBM), Random Forest (RF), and eXtreme Gradient Boosting (XGB), and one boosting algorithm: Adaptive Boosting (AdaBoost). We selected the “rbf” kernel function for SVM and set its regularization parameter “C” to 1. For DT and RF models, we used the “gini” criterion to measure the quality of each split. The number of base learners was controlled by setting the “n_estimators” parameter for GBDT, RF, AdaBoost, XGB, and LGBM, which were set to 100, 100, 50, 800, and 100, respectively. Before training, we performed feature selection to determine the most important combination of features to use as input for the machine learning models. Subsequently, we obtained predictions for all test data using the trained models.

## 3 Result

### 3.1 Statistical analysis

We evaluated the performance of our machine learning model using sensitivity and specificity. Sensitivity measures the detection ability of the model when screening positive cases, which is the proportion of true positive results to all actual positive results. The closer the sensitivity is to 1, the lower the rate of missed diagnoses for true positives. Specificity measures the ability of the model to exclude non-patients, which is the proportion of true negative objects that the model can identify when the detection target does not have autism. The closer the specificity is to 1, the lower the misdiagnosis rate. Both sensitivity and specificity are important metrics for evaluating the performance of a machine-learning model. All data analysis, model training, and evaluation were performed using Python (version 3.9.13) and Scikit-learn (version 1.2.2).

### 3.2 Comparison between different models

To compare the performance of seven different models in predicting the diagnosis of ASD and identify the model with the best classification performance, we trained various models using the same set of features. We use a total of 15 features as inputs for the 7 models we built. These features are listed in Table 3. During model training, we ensured that each model used the same amount of data for both training and testing. Among the models tested, LGBM exhibited significantly better specificity which is 0.933 than SVM, DT, AdaBoost, RF, and XGB, compared to GBDT 0.778.

**Table 3:** Comparison between different models with 15 selected features.

Model	Specificity	Sensitivity
SVM	0.889	0.889
DT	0.767	0.911
GBDT	0.778	0.967
LGBM	0.933	0.856
ADA	0.878	0.811
XGB	0.822	0.915
RF	0.856	0.878

### 3.3 Selection of the most important features

To determine and select the optimal number of important features for training, we gradually increased the number of input features from 1 to 15, adding them in order of the mRMR feature selection method described in the feature selection section. Because LGBM shows a comparative performance in Table 3, we used the LGBM model as the base classifier for this experiment.

Based on the result presented in Table 4, we observed that when using the first 9 features as inputs, the model achieved its highest sensitivity value of 0.909, while maintaining a relatively high specificity level of 0.922. As we gradually increased the number of features to 9, we noticed an overall upward trend in specificity. However, it did not continue to increase with additional features. Meanwhile, when the number of features was limited to 2 and 5, although sensitivity was high, reaching 0.900, specificity remained relatively low, failing to exceed 0.820. Furthermore, after surpassing 9 features, sensitivity could not reach above 0.900. Based on these findings, we identified the 9 best features for constructing our LGBM model, namely Q21, Q6, Q9, Q13, Q7, Q14, Q5, Q15, and Q17. Detailed descriptions of these question features are provided in the Table 5.

**Table 4:** Sensitivity and specificity at varying feature numbers from 1 to 15 on LGBM.

Number of features	Specificity	Sensitivity
1	0.817	0.744
2	0.904	0.833
3	0.911	0.813
4	0.926	0.836
5	0.904	0.865
6	0.902	0.787
7	0.878	0.796
8	0.896	0.769
9	0.922	0.909
10	0.878	0.833
11	0.891	0.833
12	0.863	0.823
13	0.833	0.771
14	0.933	0.856
15	0.933	0.856

**Table 5:** The 9 best features selected to construct LGBM model.

Rank	Question number	Question descriptions
1	Q21	Does your child understand what people say?
2	Q6	Does your child ever use his/her index finger to point, to ask for something?
3	Q9	Does your child ever bring objects over to you (parent) to show you something?
4	Q13	Does your child imitate you? (e.g., you make a face; will your child imitate it?)
5	Q7	Does your child ever use his/her index finger to point, to indicate interest in something?
6	Q14	Does your child respond to his/her name when you call?
7	Q5	Does your child ever pretend, for example, to talk on the phone or take care of dolls, or pretend other things?
8	Q15	If you point at a toy across the room, does your child look at it?
9	Q17	Does your child look at things you are looking at?

## 4 Discussion

### 4.1 Interpretation of result

**Figure 3:**
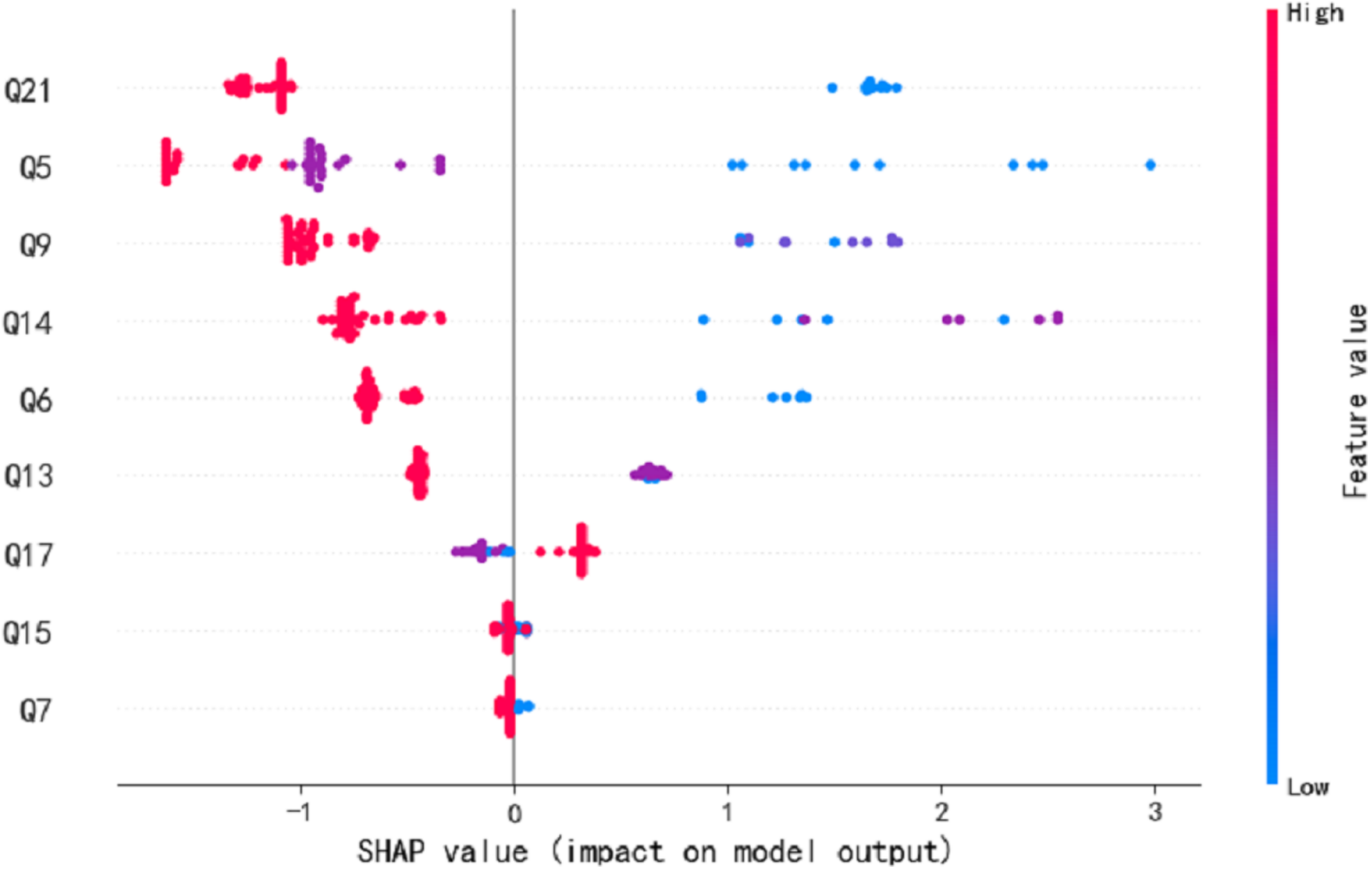
The SHAP summary plots for the LGBM model. This depicts the 9 most effective features on prediction.

**Figure 4:**
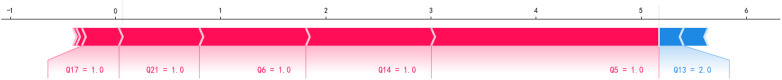
SHAP force plot for one child of the test set.

We employed seven commonly used machine learning techniques to model early screening models for Autism. The experimental results demonstrated that the LGBM model outperformed the other models, exhibiting superior specificity and sensitivity. Notably, the LGBM model achieved its highest sensitivity value of 0.909 when the number of features reached 9 while maintaining a consistently high specificity level of 0.922. We analyzed our autistic patients and individual patient explanations using SHAP technology. Figure 3 presents SHAP summary plots of the top 15 clinical features in the contribution of ML models to the prediction of autism development in our study. The implication is that the model’s interpretation is consistent with the feature rank ordering we obtain via mRMR. Figure 4 shows a patient at low risk of having autism.

### 4.2 Clinical implications

After optimizing the feature for convenience in clinical practice, the time required is reduced, making it easier for parents to complete and for professionals to assess. The consistency in clinical presentation is evident in the construction of 9 items based on the MRMR model. Among these, questions 5, 7, 9, 13, and 15 are core components of the CHAT-23 clinical scale. These items cover areas such as pretend play, pointing skills, initiating sharing, imitation ability, and joint attention. The lack or delay in these specific skills reflects the clinical characteristics of social aspects in children with autism spectrum disorders.

### 4.3 Limitations

While building machine learning models can provide significant assistance for early autism detection, there are still some limitations regarding practical application. Firstly, our machine learning models are specifically tailored to data from Chinese children, meaning they perform best within this demographic. Exploring the development of more universally applicable models might be a worthwhile direction for future research. Additionally, once the models are built, their parameters remain static, making them outdated over time. Currently, our best model uses at least nine features, further reducing the number of features used in building machine learning models could make them more efficient in practical applications.

### 4.4 Future research directions

Our future goal is to combine the model with an online questionnaire format and deploy it on a web platform. By using fewer questions, we can quickly provide accurate autism assessment results to parents via mobile phones or other online tests, enabling them to obtain efficient and reliable references for their children. This approach not only improves efficiency but also reduces the workload of medical personnel.

## 5 Conclusions

In this study, we have developed a machine learning approach based on the Chinese children’s scale data, which was obtained by filling in the CHAT-23 questionnaire questions. Then, we conducted feature engineering by using statistical methods and mRMR feature selection on the 23 questions of the CHAT-23 scale. Through the above process, we ranked these questions according to their importance, and the final 9 questions were selected, specifically Q21, Q6, Q9, Q13, Q7, Q14, Q5, Q15, and Q17. These selected questions were then applied to an LGBM model, which demonstrated superior performance compared to the other popular machine learning models (SVM, DT, GBDT, ADA, XGB, RF). By utilizing this machine learning model with only 9 questions, we achieved comparable results, which use all 23 questions as features for computer-aided autism diagnosis.

## Data Availability

The original contributions presented in the study are included in the article material, further inquiries can be directed to the corresponding author.
